# The complete mitochondrial genome of the Spotted Greenshank *Tringa guttifer* (Charadriiforemes: Charadriidae)

**DOI:** 10.1080/23802359.2019.1629349

**Published:** 2019-07-12

**Authors:** Wei Liu, Yuxiao He, Jingjing Ding, Qing Chang

**Affiliations:** a Ministry of Ecology and Environment of the People's Republic of China, Nanjing Institute of Environmental Sciences, Jiangsu, People’s Republic of China;; b Jiangsu Key Laboratory for Biodiversity and Biotechnology, College of Life Sciences, Nanjing Normal University, Nanjing, Jiangsu, People’s Republic of China;; c Jiangsu Academy of Forestry, Nanjing, Jiangsu, People’s Republic of China

**Keywords:** *Tringa guttifer*, mitochondrial genome, phylogenetic analysis

## Abstract

The complete mitochondrial DNA genome of the Endangered Spotted Greenshank, *Tringa guttifer*, was detected using phenol–chloroform extraction procedure and polymerase chain reaction method. It is a circular molecule of 16,935 bp in size, exhibits the typical structure of mitochondrial genomes of Charadriiformes, containing 13 protein-coding genes, 2 rRNA genes, 22 tRNA genes, and a control region. Overall base composition of the complete mitochondrial DNA is A (31.7%), T (25.5%), C (29.5%), and G (13.3%), the percentage of A and T (57.2%) is higher than G and C. The phylogenetic relationships of 34 species were reconstructed using the maximum-likelihood and Bayesian inference, and unravel *T. guttifer* is close to *T. semipalmata*.

The Spotted Greenshank (*Tringa guttifer*) is a small shorebird, breeding around the Russian Far East and wintering in the southeast Aisa (Zöckler et al. 2018). Due to the loss or degradation of wetland habitats and human activity (Melville et al., 2016; Yu et al. 2019), the global population of *T. guttifer* was roughly estimated at no more than 1300 individuals, and the species is facing a continuing decline (BirdLife International 2017). Based on the current knowledge of the Spotted Greenshank, the species is listed as ‘Endangered’ by IUCN and a Class II protected species under China’s National Wildlife Law. Although sparse surveys have introduced the Spotted Greenshank ecology, the studies regarding to the genetic background of *T. guttifer* scarce up to now.

In this study, the complete mitochondrial genome sequence of *T. guttifer* was determined and deposited into GenBank (GenBank accession number: MK905885). The samples were collected when we banded migratory shorebrids in the eastern coast of Jiangsu province, China. Feather and blood sample was preserved with 90% ethanol in sterilized centrifuge tube and stored at –20 °C in the Nanjing Normal University (specimen voucher number: NJNU-TG1801). The genomic DNA was isolated by the standard phenol–chloroform extraction procedure (Sambrook and Russell 1989).

After proofread and assembled using Seqman Pro (DNASTAR, Inc., Madison, WI), the complete mtDNA genome has been obtained. The circular mitogenome is 16,935 bp in length, containing 13 protein-coding genes (PCGs), 22 transfer RNA (tRNA) genes, 2 ribosomal RNA (rRNA) genes, and a control region. The mitochondrial gene arrangement of *T. guttifer* was identical to other species of Charadriiformes (Hu et al., 2017). The overall base composition of the *T. guttifer* mitogenome was biased toward A + T (57.2%), and 31.7% A, 25.5% T, 29.5% C, and 13.3% G.

In order to unravel the phylogenetic relationship of *T. guttifer* exactly, two species from Phasianidae were selected as outgroup. Moreover, 31 extra complete mitochondrial genomes from Charadriiformes were downloaded to construct the phylogenetic tree with *T. guttifer*. The phylogenetic tree was inferred under the maximum-likelihood (ML) and Bayesian inference (BL), which performed with RAxML 8.2.4 and MrBayes 3.2.2 respectively. The consensus tree demonstrates the spotted greenshank is closely related to *T. semipalmata* with high support (Figure 1), and the relationships of the genus *Tringa* based on the nearly complete mtDNA strongly support monophyly of the genus *Tringa.*

**Figure 1.**
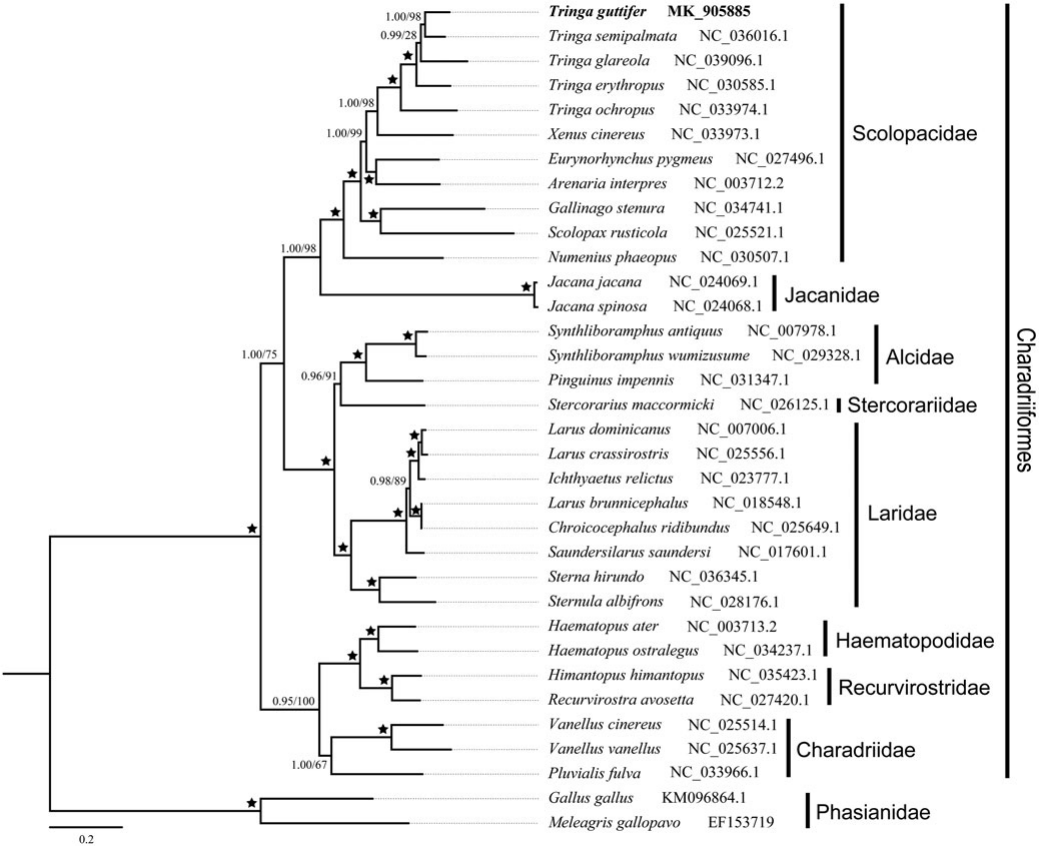
Phylogenetic trees based on the complete mitogenomes of 34 species. Numbers above the tree branches are the posterior probabilities and ML bootstrap support. GenBank accession numbers are given after the species name. Bold letters represent the sequence in this study.
